# Transfusion-Associated Lyme Disease – Although Unlikely, It Is Still a Concern Worth Considering

**DOI:** 10.3389/fmicb.2018.02070

**Published:** 2018-09-04

**Authors:** Charles S. Pavia, Maria M. Plummer

**Affiliations:** ^1^Department of Biomedical Sciences, New York Institute of Technology College of Osteopathic Medicine, New York, NY, United States; ^2^Department of Clinical Specialties, New York Institute of Technology College of Osteopathic Medicine, New York, NY, United States

**Keywords:** lyme disease, *Borrelia burgdorferi*, hematogenous spread, blood donor, blood transfusion, diagnostic testing, diagnostic issues

## Abstract

Even though hematogenous spread of the Lyme disease spirochete, *Borrelia burgdorferi*, has been well documented, and there are more than 300,000 cases per year of Lyme disease in the United States, no evidence (anecdotal or published) of transfusion-associated Lyme disease has been reported. Such a possibility would seem to exist but various factors, as discussed in this perspective, make this less likely to occur. Nonetheless, if not done already, safeguards need to be put in place at blood collection and dispensing facilities, possibly with the assistance of diagnostic microbiology and immunology laboratories, to ensure that the potential for the transfer of the Lyme disease spirochete through a blood transfusion remains a theoretical consideration rather than a real possibility.

## Introduction

The most common tick-borne disease in the United States is Lyme disease (Nelson et al., 2015) yet, so far, there have been no reports of transfusion-associated infection with the causative agent, *Borrelia burgdorferi*. This perspective explains the reasons why such an occurrence would seem unlikely although proper monitoring of donated blood still needs to be done, in this regard, in order to keep the blood supply Borrelia-free. According to statistics kept by the United States Centers for Disease Control and Prevention (Centers for Disease Control and Prevention [CDC], 2017), the CDC has received notifications each year, for the past 10 years, by state health departments and the District of Columbia, that approximately 25,000 to 30,000 confirmed cases of Lyme disease occur annually. However, it has been subsequently reported by the CDC (Nelson et al., 2015) that this number is not reflective of every case of Lyme disease that probably occurs in the United States every year. Indeed, as a prelude to this published report, the CDC announced, in the summer of 2013, that the actual incidence of physician-diagnosed Lyme disease was much higher with the number of cases estimated at 300,000 per year. Lyme disease occurs in certain geographically distinct sections of the continental states of the United States and in several select countries worldwide, especially in Europe. More specifically, in North America it has a limited endemicity occurring primarily in: (i) the northeastern Atlantic coastal areas from Maine to Maryland, (ii) the Midwestern states Wisconsin and Minnesota, and (iii) certain areas of the far western states of California, Oregon and Washington. The geographic distribution of Lyme disease closely follows the location of the arthropod vector (the so-called deer tick) of Lyme disease in the United States — *Ixodes scapularis* (previously called *I. dammini*) in the eastern and Midwestern regions, and *I. pacificus* in the far west.

Lyme disease is an illness which can present in a wide array of clinical manifestations (Pavia, 2015) that consist of: (i) a unique erythematous rash that expands peripherally from an area having central clearing; (ii) non-specific symptoms consisting of headache, fever, neck stillness and nausea; (iii) neurologic abnormalities, most commonly facial nerve (Bell’s) palsy, and meningitis; and (iv) arthritis, especially in the knee, in about 50–60% of untreated patients. Additional dermatologic abnormalities, not typically seen in North America, can occur in Lyme disease patients residing in Europe. Patients develop these symptoms most frequently during the spring to early autumn months when both nymphal and adult stage ticks are present in large numbers and are most active, and people, especially children, take part in many outdoor, and recreational activities. Soon after (ranging from about 3–32 days) a person is bitten by an *Ixodes* tick bearing the etiologic agent, *B. burgdorferi* (a spirochetal bacterium), Lyme disease typically begins with a localized dermatologic infection that leads to the development of the notable skin rash, erythema migrans (EM). During this early infectious stage, the EM rash typically becomes a uniformly expanding macule or papule starting from the center of the bite with a uniquely appearing area of central clearing and reddening with a flat red outer border somewhat resembling erythema marginatum, or southern tick-associated rash infection (Pavia and Plummer, 2017). It is important to point out, however, that when many Lyme disease patients seek medical attention, they either do not remember being exposed to, or bitten by, a tick, or a rash did develop but it went unnoticed, especially if it was very transient. It is also possible that, in rare cases, the typical EM rash never developed. In addition, during this initial phase of the infection, some patients will go on to develop certain symptoms similar to the common flu, such as fever, headache, malaise, neck stiffness, and joint pain and discomfort. In rare cases, multiple EM-like lesions can occur which result from hematogenous spread of the organism through the subcutaneous capillaries. The serious systemic complications of Lyme disease may include migratory and polyarticular arthritis, neurologic involvement with cranial nerve (7th) palsies, radiculopathy and meningitis, and certain cardiac abnormalities such as myocarditis, and arrhythmias. The beneficial effects of penicillin or tetracycline, used to treat the initial, early cases that were described in the late 1970s, implicated a newly described disease having a microbial origin, with the likely pathogen being a bacterium. This illness, before it became known as Lyme disease or Lyme borreliosis, was initially given the designation of Lyme arthritis with the etiologic agent subsequently proven to be a spirochete having the designated name of *B. burgdorferi.*

## Discussion

Given the backdrop of our preceding introductory comments, we will now expand on several of the key factors that were put forth initially in the informative article by Ginzburg et al. (2013), and provide a more in-depth analysis, pertaining to various other factors that could play a role on the occurrence or lack of transfusion-associated Lyme disease. As with other spirochetal diseases, such as syphilis (Orton, 2001), it is believed that the spread of *B. burgdorferi* bacteria from the EM rash to the various extracutaneous sites occurs primarily through the hematogenous route. Based on studies done, in part, by us (Wormser et al., 2001, 2005; Pavia and Plummer, 2013) and in collaboration with others, the presence of live blood-borne *Borrelia* in the peripheral vasculature has been well documented in adult patients with EM who had not yet received appropriate antibiotic treatment. Such findings have generated some concern over whether or not blood transfused from a spirochetemic donor might unwittingly transfer Lyme disease to a recipient in need of whole blood or any of its component parts (Ginzburg et al., 2013). As pointed out by these authors, it would seem logical to assume that Lyme disease patients would be too symptomatic to want to donate blood and thus it would be highly remote that blood contaminated with *B. burgdorferi* would be available for human use. On the other hand, it has been shown in one clinical study (Wormser et al., 2001) that 11% of 93 patients with *B. burgdorferi* in their bloodstream did not present with any objective clinical symptoms except for having the EM rash, and clinical experience (as mentioned above) suggests that for some patients the EM rash may go unrecognized. Thus, as suggested further by Ginzburg et al. (2013), it is entirely plausible that spirochetemic patients with an unrecognized EM, or one of shortened duration, might present themselves as potential donors. In addition, it has been shown (Wormser et al., 2001) that, with such patients, *Borrelia* are likely to be found circulating in the blood sporadically or they may persist for a time period ranging from 2 to 5 weeks and in some cases beyond this time frame. On the other hand, when certain extracutaneous disease manifestations such as those involving the musculoskeletal, neurologic and cardiovascular systems occur in *B. burgdorferi*-infected patients, they typically develop during the one to 2 month time frame after a tick bite. Under these circumstances, spirochetemia would not be expected unless the patient concurrently displayed the EM rash (Nowakowski et al., 2009), but they too would be unlikely candidates as blood donors. Also, along these lines, some of our more recent related studies (Pavia and Plummer, 2013), done in mice, have shown that intermittent spirochetemia, with the presence of infectious/pathogenic *Borrelia*, occurs during early *B. burgdorferi* infection, which could be reflective of what takes place during human Lyme disease.

It has been well documented that various pathogens, such as the syphilis spirochete, *Treponema pallidum*, and the spirochetal bacterium *Borrelia recurrentis*, the protozoans *Babesia* and *Plasmodia*, and several viruses, can cause transfusion-transmitted infection (Wang and Lee, 1936; Kone et al., 1989; Orton, 2001; Herwaldt et al., 2011; Seed et al., 2015). Although transfusion-associated Lyme disease has not yet been reported, another *Borrelia* species – the afore-mentioned and related *B. recurrentis* – has been shown to cause transfusion-associated relapsing fever (Wang and Lee, 1936). In this context and as delineated in detail by Ginzburg et al. (2013), these microorganisms share several common elements including (i) an initial pro-dromal infectious phase having little or no clinical manifestations in the prospective blood donor; (ii) the pathogen remains alive and virulent during the handling, processing and short-term storage of the blood sample; and (iii) there must be a recipient population that is susceptible to developing Lyme disease or any of these other infectious disorders following transfer of a contaminated blood product from a donor who had not received antibiotic treatment.

Currently used screening procedures appear to be effective for eliminating (or nearly eliminating) blood transfusion cases of a wide variety of infectious diseases with the most prominent being syphilis, hepatitis B and HIV infections (Orton, 2001; Centers for Disease Control and Prevention [CDC], 2011). Potential donors having a confirmed diagnosis of Lyme disease or who report being bitten by a tick are usually deferred for a certain time period at some of the American blood collecting facilities. However, no serologic testing or molecular analyses are routinely done which would be needed in order to fully ensure that potential asymptomatic, *Borrelia*-infected, donors are excluded from the donor pool (Ginzburg et al., 2013). Furthermore, a significantly low number of approximately 25% of *B. burgdorferi*-infected patients with early disease remember being exposed to ticks (Tibbles and Edlow, 2007); thus, the inability for a tick bite to be noticed would not technically prevent an infectious person from donating blood. It is therefore possible that at least a small percentage of symptom-free persons, who are infected with *B. burgdorferi*, might still be viewed as acceptable donor candidates based on not having been detected by currently available diagnostic testing systems (Ginzburg et al., 2013). What role then should the clinical microbiology or immunology laboratory play under these circumstances? Most of these diagnostic facilities would be expected to aid blood collection agencies in being able to identify prospective blood donors who may be infected with any of the previously cited pathogens. However, diagnostic testing with regards to Lyme disease can be problematic especially during early stage disease where several studies, including our own (Pavia et al., 2000) and reviewed by Aguero-Rosenfeld et al. (2005), have shown that <50% of patients with EM, some of which are culture-confirmed, are seronegative for anti-*B. burgdorferi* antibodies. It is noteworthy that many of these patients do turn seropositive, several weeks later, when convalescent sera are tested for antibodies, even after curative antibiotic treatment has been given. Improvements toward developing serologic-based assays having better sensitivity without compromising specificity are currently in the developmental stage (Branda et al., 2018) which include using presumed immunodominant recombinant borrelial proteins or synthetic peptides. An additional diagnostic platform, the utilization of molecular-based detection methods, has focused mainly on PCR-based techniques (Aguero-Rosenfeld et al., 2005), but it too has its limitations. Similar to other pathogen-related nucleic acid amplification tests, molecular testing for Lyme disease is not done routinely or has yet to be fully accepted by most diagnostic laboratories, due primarily to poor sensitivity.

Another key related finding has shown that *B. burgdorferi* can be transferred from spirochetemic donor mice to naive recipients during an experimental blood transfusion (Gabitzsch et al., 2006) that closely mimicked typical human blood transfusion procedures. However, to date, such findings have not been reported using human blood that has been mixed *in vitro* with a pathogenic strain of *B. burgdorferi* to determine whether an infection can be transferred at various time intervals after the mixtures have been held under blood-storage conditions. Similarly, there are no published data on whether spirochetemic blood from Lyme disease patients can cause an infection, or disease manifestations after being transferred into a recipient host. Obviously, ethical considerations preclude transferring potentially infectious human blood to healthy human volunteers, in an experimental study, in order to resolve this issue. Alternative studies could be performed, however, that would provide useful information based on using the well characterized mouse model for Lyme disease. In this case, human blood containing live spirochetes and subjected to blood-banking storage conditions, similar to what we have reported before (Nadelman et al., 1990; Pavia and Plummer, 2013) would be injected into mice and then the mice would be evaluated for an active infection by using standard techniques, such as measuring seroconversion, and performing culture and histopathology of key target organ or tissue sites. This proposed study would follow closely a related recently published report (Thorp and Tonnetti, 2016) which showed that the closely related relapsing fever spirochete, *Borrelia miyamotoi*, was able to survive and infect mice after being kept under standard storage conditions with human blood or most of its component parts, suggesting that transmission by blood transfusion of this pathogen was possible. Other additional related studies, such as those proposed by others (Ginzburg et al., 2013), would involve transfusing spirochetemic blood from infected mice into uninfected mice after the blood had been processed and stored under various blood-banking storage conditions, but this might have less impact and relevance than performing the aforementioned experiments involving the use of human blood and recipient mice.

**FIGURE 1:**
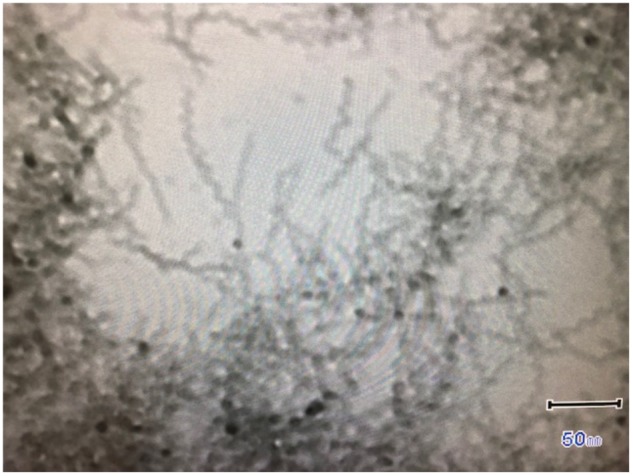
Photomicrograph of Giemsa-stained *B. burgdorferi*, sensu stricto, after culture in BSK medium as previously described (Pavia and Plummer, 2013). Magnification, 400X; bar = 50 μ.

**FIGURE 2:**
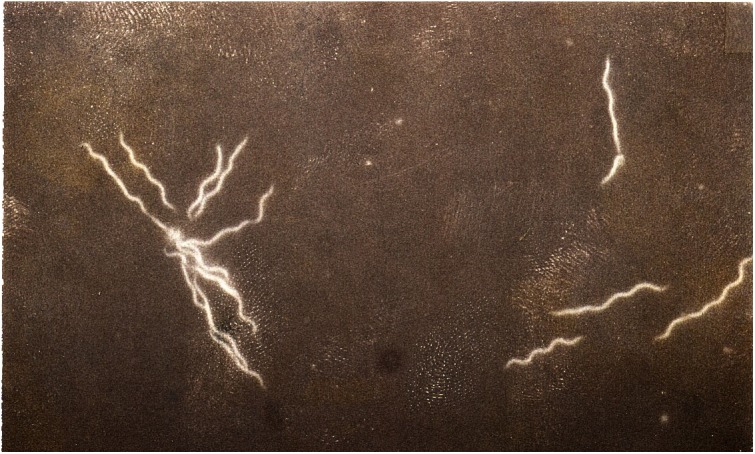
Photomicrograph of a wet mount preparation of a small growing colony (left side) and separate organisms of *B. burgdorferi* after several days of culture in BSK medium (Wormser et al., 2001); dark-field microscopy, 400X magnification.

Other factors to consider on whether or not transfusion-associated Lyme disease could become a significant problem pertain to the microbiologic properties of the etiologic agent. Similar to other pathogenic spirochetes, *B. burgdorferi* (**Figures 1**, **2**) is a somewhat fragile organism having fastidious *in vitro* growth requirements while the syphilis spirochete had remained non-culturable (Pavia, 2015) for over a century, until very recently, when its continuous *in vitro* growth and replication was finally achieved (Edmondson et al., 2018). Borrelia are also somewhat difficult to identify microscopically using simple staining techniques such as the Gram stain, but they can be stained with Giemsa (**Figure 1**), or visualized using dark-field or phase-contrast microscopy (**Figure 2**). In this regard, the published literature (Baranton and Saint-Girons, 1987; Badon et al., 1989; Nadelman et al., 1990) has shown a certain amount of variability on the time-dependent survivability of *B. burgdorferi in vitro* when mixed with different blood products and then when stored under blood-banking conditions, but the maintenance of borrelial infectivity and pathogenicity has never been analyzed under such conditions. Additionally, several studies (Schwan and Piesman, 2000; Grimm et al., 2004; Tilly et al., 2006) have delineated nicely the interrelationship between the synthesis and expression of some of the outer surface proteins (OSP), especially OspA and OspC, of *B. burgdorferi* in terms of their respective roles in borrelial infectivity and pathogenesis. The key developmental phases of these proteins are distinct depending upon whether *B. burgdorferi* resides in the tick vector or in the mammalian host. While in the tick, OspA is upregulated yet, during and soon after transmission to a victim via a tick bite, its expression gradually decreases. Then, OspC gets turned on and is associated with the establishment of an active infection. However, some exceptions to this scenario have been reported (Tilly et al., 2009), whereby a wild-type mutant of *B. burgdorferi* which lacked OspC has been shown to give rise to a persistent infection in experimentally challenged mice. Also, along these lines, it has been reported that, based on the analysis of patient isolates (Wormser et al., 1999), there are at least three genetic subtypes of *B. burgdorferi* that could influence the pathogenesis of Lyme disease. Interestingly, a specific subtype was found to be associated with hematogenous spread more so than the other two subtypes. Taken together, these findings illustrate the complexity of the Lyme disease bacterium after it enters the human host which could impact on the immune system’s response and control of the infection, as well as on the transmissibility of potentially infectious blood from one person to another leading to a symptomatic post-transfusion infection.

## Conclusion

In conclusion, it would seem logical and safe to assume that transfusion-associated Lyme disease would be an unlikely occurrence given the belief that symptomatic patients, the potential likely source of spirochetemic blood, would be too ill to want to donate blood or would be rejected by standard or routine screening practices, if they tried to. Nonetheless, because our knowledge in this area is still limited, additional studies are warranted to evaluate various factors associated with Borrelia-human blood interactions, especially in light of some of the unique genetic and microbiologic properties of the pathogen, in order to determine more fully the potential for *B. burgdorferi* to cause transfusion-transmitted Lyme disease. In addition, it is necessary to consider determining what the optimal criteria and policies should be, such as the appropriate use of approved diagnostic methods, for monitoring blood products for possible contamination with the Lyme disease spirochete, especially in geographic areas in which *B. burgdorferi* infection and other related tick-borne diseases are endemic.

## Author Contributions

CP and MP wrote the manuscript. CP performed the cultures mentioned in the two figures and MP assisted in preparing the photomicrographs.

## Conflict of Interest Statement

The authors declare that the research was conducted in the absence of any commercial or financial relationships that could be construed as a potential conflict of interest.
